# Implications of Pyrosequencing Error Correction for Biological Data Interpretation

**DOI:** 10.1371/journal.pone.0044357

**Published:** 2012-08-30

**Authors:** Matthew G. Bakker, Zheng J. Tu, James M. Bradeen, Linda L. Kinkel

**Affiliations:** 1 Department of Plant Pathology, University of Minnesota, St. Paul, Minnesota, United States of America; 2 Supercomputing Institute, University of Minnesota, Minneapolis, Minnesota, United States of America; Argonne National Laboratory, United States of America

## Abstract

There has been a rapid proliferation of approaches for processing and manipulating second generation DNA sequence data. However, users are often left with uncertainties about how the choice of processing methods may impact biological interpretation of data. In this report, we probe differences in output between two different processing pipelines: a de-noising approach using the AmpliconNoise algorithm for error correction, and a standard approach using quality filtering and preclustering to reduce error. There was a large overlap in reads culled by each method, although AmpliconNoise removed a greater net number of reads. Most OTUs produced by one method had a clearly corresponding partner in the other. Although each method resulted in OTUs consisting entirely of reads that were culled by the other method, there were many more such OTUs formed in the standard pipeline. Total OTU richness was reduced by AmpliconNoise processing, but per-sample OTU richness, diversity and evenness were increased. Increases in per-sample richness and diversity may be a result of AmpliconNoise processing producing a more even OTU rank-abundance distribution. Because communities were randomly subsampled to equalize sample size across communities, and because rare sequence variants are less likely to be selected during subsampling, fewer OTUs were lost from individual communities when subsampling AmpliconNoise-processed data. In contrast to taxon-based diversity estimates, phylogenetic diversity was reduced even on a per-sample basis by de-noising, and samples switched widely in diversity rankings. This work illustrates the significant impacts of processing pipelines on the biological interpretations that can be made from pyrosequencing surveys. This study provides important cautions for analyses of contemporary data, for requisite data archiving (processed vs. non-processed data), and for drawing comparisons among studies performed using distinct data processing pipelines.

## Introduction

Current DNA sequencing capacity offers the opportunity to study microbial communities in unprecedented detail. However, quality standards often lag behind technical innovation and many early studies of microbial communities using second generation sequencing now appear to have substantially overestimated microbial diversity [1]. An expanding list of criteria has been proposed to screen out low quality reads from pyrosequencing datasets [2], [3], but these have not proven adequate to eliminate spurious diversity. Although high sequencing accuracy can be achieved by removing reads that are most likely to contain errors, low error rates may still accumulate to substantial effect in datasets with hundreds of thousands (or more) sequence reads. Furthermore, data processing methods can continue to introduce error after sequencing is completed, as with imperfections in multiple sequence alignment during the process of defining operational taxonomic units (OTUs) [4].

One approach to dealing with the problem of sequence error has been to simply shed detail from a dataset until there is a high probability that the influence of PCR or sequencing errors has been removed, for example with the use of broad criteria for delimiting OTUs or in approaches that discard all of the least-frequently occurring sequence variants [5]. A preferable approach is to devise means of identifying and correcting errors such that accurate detail can be maintained. The AmpliconNoise program [6] was reported as such a method for pyrosequencing error detection and correction and was quickly incorporated into the major processing pipelines for pyrosequencing data [7], [8]. However, users are often uncertain how the interpretation of their data will be affected by the choice of different processing methods. A great deal of the effort given to evaluating sequence-based methodologies has been given to reducing OTU inflation and forming the correct number of OTUs. However, most experimental studies aim to do much more than derive simple richness estimates, and the interpretive impacts of a choice of data processing pipeline are likely to be broader than this single, predominant criterion. Compared to simple constructed communities often used in evaluating new methods, there are many more opportunities for interpretations to shift when the work concerns a real dataset derived from a complex experiment in a natural environment. Here, we give careful consideration to interpretive implications of the AmpliconNoise processing method on an original dataset of soil microbial community samples.

**Table 1:** Number of sequences failing quality screening criteria and total number of sequences remaining (bold, italics), for standard processing pipeline and for AmpliconNoise processing.

Screening Criteria	Standard Output	AmpliconNoise Output
***Initial***	***409,997***	***232,792***
<100% match to 5' primer	31,948	NA
Sequence length <120 bp	137,753	NA
Ambiguous bases present	6,295	0
Homopolymers >6 bases	149	12
Avg Qscore <25	8,985	NA
Poorly aligning to database	138	106
***Remainder after 1st stage screening***	***253,973***	***232,674***
***Uniques***	***22,351***	***3,053***
***Pre-cluster sequences differing by 1 bp***	***10,529 uniques***	***NA***
Flagged as chimeras	1,081	1,460
Phyla other than target	25	25
***Net sequence read yield***	***252,867***	***231,189***
***Sequence reads per sample (+/−SE)***	***4,214+/−101***	***3,853+/−99***
***Unique sequence reads***	***10,250***	***2,751***
***OTUs***	***1,166***	***792***
***OTUs containing shared reads***	***769***	***739***
**Equalize sampling effort (subsample to 3,000 reads per sample)**		
***OTUs per sample (+/−SE)***	***112+/−2***	***120+/−2***

The sum of sequences failing each criterion in the initial screening is greater than the number of sequences dropped because some sequences failed on multiple criteria. AmpliconNoise processing includes a test of matching to the 5' primer, and does not make use of quality scores. OTUs were defined based on a 3% sequence dissimilarity threshold, using the average neighbor method.

## Materials and Methods

Soil sampling was performed at the Cedar Creek Ecosystem Science Reserve (CCESR; part of the National Science Foundation Long-Term Ecological Research network) in July of 2009, from a long-term plant richness manipulation [9]. We targeted soil under the dominant influence of each of four different plant species (two C4 grasses: *Andropogon gerardii*, *Schizachyrium scoparium*; two legumes: *Lespedeza capitata*, *Lupinus perennis*) by collecting soil cores from the base of individual plants. Each sample consisted of four bulked soil cores (5 cm diameter, 30 cm depth) collected from different individuals within the same plot and homogenized by hand. Each plant species was sampled in five different plant richness treatments (monoculture and assemblages of 4, 8, 16 or 32 species). There were three plot-level replicates per host-plant richness combination.

The PowerSoil DNA kit (MO BIO; Carlsbad, CA USA) was used to extract DNA from soil. The manufacturer’s protocol was modified with extended bead beating and sonication to enhance recovery of DNA from Actinobacterial spores [10]. Selective primers were used to amplify a portion of the 16s rDNA gene. We used StrepB [11] as our forward primer, and the reverse complement of Act283 [12] as our reverse primer, each at a final concentration of 200 nM. Both primers are selective for Actinobacteria and together amplify a fragment of approximately 165 nucleotides, encompassing the V2 variable region of the 16S rRNA gene. Primers were modified to contain one of 30 different 10mer identifying barcodes [13]. PCRs consisted of 10 ng of template DNA in a 50 uL reaction volume using PCR Supermix High Fidelity (Invitrogen; Carlsbad, CA USA). PCR conditions consisted of an initial denaturation step of 30 sec at 94 C, followed by 30 cycles of 30 sec 94 C, 30 sec 57 C, 60 sec 70 C. Products of PCRs were passed through the QIAquick PCR Purification Kit (Qiagen; Valencia, CA USA), quantified by spectrophotometry, diluted with elution buffer to approximately 15 ng/uL, and quantified by fluorometry (Quant-iT dsDNA HS assay kit; Invitrogen). Thirty samples, each with a unique primer barcode, were combined in equimolar amounts to form each of two pooled amplicon samples. Emulsion PCR and sequencing were performed using a GS FLX emPCR amplicon kit according to the manufacturer’s protocols (454 Life Sciences; Branford, CT USA). Each pooled sample was run on one region of a picotitre plate on the GS FLX sequencing system [14] at the University of Minnesota BioMedical Genomics Center. Resulting sequence data have been submitted to the NCBI Sequence Read Archive as accession SRA019985.3.

Sequence data were processed through the program AmpliconNoise, version 1.24 [6] for the detection and correction of probable errors. The dataset was processed on a per sample basis, with the raw flowgram signals as the input to AmpliconNoise. Initial processing tested for a perfect match to the forward primer, truncated flowgrams at 225 flows and discarded any reads that did not reach this length threshold. The PyroNoise algorithm was run with parameters set at s = 1/60, c = 0.01. The SeqNoise algorithm was run with parameters set at s = 1/30.3, c = 0.08.

**Figure 1:**
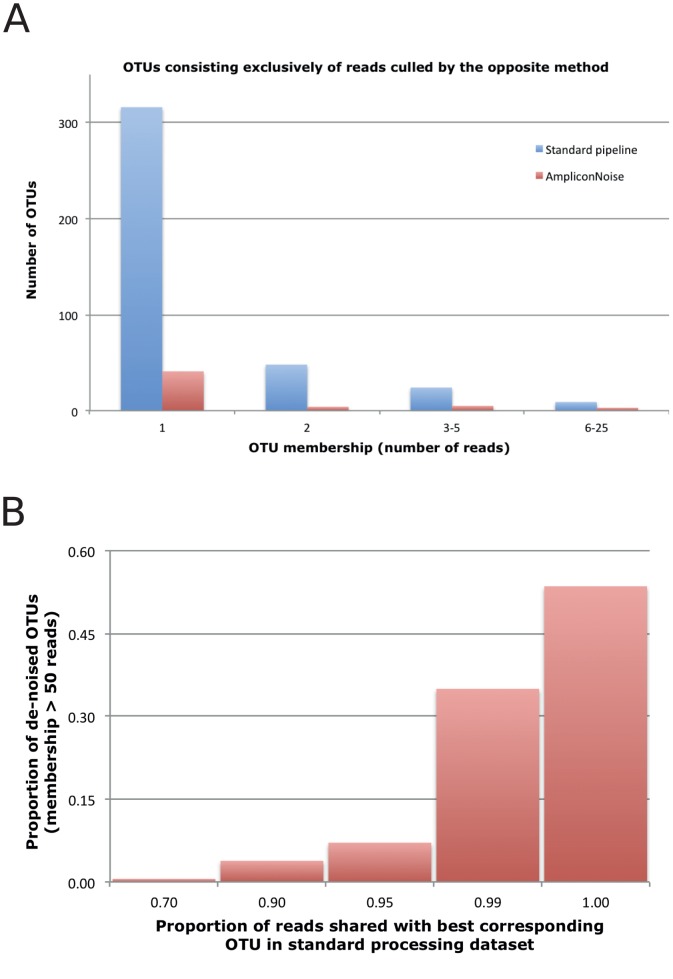
Corresponding OTUs between data processing methods. A) Each method generated some OTUs which consist entirely of sequence reads that were culled in the alternate method. These inconsistent OTUs were mostly singletons and were more abundant in the standard pipeline. **B)** Most OTUs had a clear corresponding OTU in the alternate method. Data shown are OTUs having a membership>50 reads in the AmpliconNoise dataset, and the proportion of the membership of each OTU that was shared with the best corresponding OTU in the standard pipeline dataset.

Subsequent processing, and all processing for the standard (not-denoised) pipeline, was done with the program Mothur 1.20.1 [8]. Quality screening criteria and the number of reads culled are shown in Table 1. Sequences were aligned to the Silva reference database [15] using kmer searching with a ksize of 8 to find the best template sequence and the gotoh alignment method with a reward of +1 for a match and penalties of −1 for a mismatch, −2 for opening a gap, and −1 for extending a gap. Aligned sequences were truncated to a length of 150 nucleotides and screened for chimeras using the UChime method [16]. In the standard processing pipeline, sequences that differed by only a single base pair were pre-clustered. Sequences passing these quality criteria were clustered into operational taxonomic units (OTUs) using a 3% sequence dissimilarity criterion and the average neighbor clustering method. We rarefied to a consistent sampling effort of 3,000 reads per sample prior to calculating diversity statistics. Five of the 60 samples were excluded from diversity analysis because they consisted of fewer than 3,000 reads. For phylogenetic diversity analysis, dendrograms were generated from sequence distance matrices using Clearcut [17], as implemented in Mothur.

The per nucleotide error rate implied by AmpliconNoise processing was calculated as the pairwise distance between the input and output sequences, where distance is defined as the number of base differences between the two sequences divided by the length of the shortest sequence, where terminal gaps are ignored and each internal gap contributes a length of one. Pairwise alignments were made with ClustalW [18] and distance was calculated using Mothur. We generated a PostgreSQL database to map reads from the raw data through AmpliconNoise processing steps and associated accession number changes, in order to contrast OTU composition between processing pipelines.

**Figure 2:**
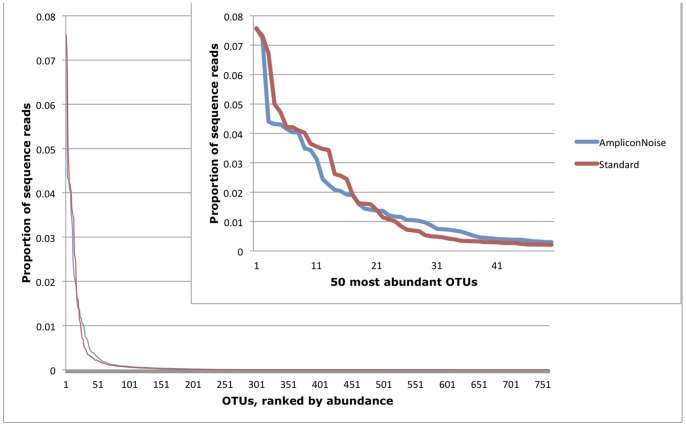
Impacts of de-noising on the rank-abundance distribution of OTUs. AmpliconNoise processing significantly altered the OTU rank-abundance distribution (two-sample Kolmogorov-Smirnov test; D = 0.20, p<0.0001), and increased evenness.

## Results and Discussion

### Quality Screening

In the standard processing pipeline, error-prone and low-quality sequences are culled from the dataset. Pre-processing in AmpliconNoise also results in sequence culling, but the actual de-noising steps correct probable errors while retaining those modified sequence reads. The subset of sequence reads culled by the two methods overlapped substantially, but the standard pipeline had a higher net yield (Table 1; net sequence reads per sample 4,214 for the standard pipeline vs. 3,853 for AmpliconNoise; t_paired_ = 6.50, p<0.01). Only 0.74% of reads passing through AmpliconNoise did not also pass through the standard pipeline. This suggests a quite modest potential for AmpliconNoise to salvage reads that would otherwise be discarded. In contrast, 9.3% of reads passing through the standard pipeline were culled by AmpliconNoise.

De-noising with AmpliconNoise will have multiple impacts on the final dataset: not only are some reads changed to correct probable errors, but the selection of reads that pass through quality screening will also be impacted. It is difficult to isolate the effects of de-noising versus effects of other quality screening criteria. For instance, length screening in AmpliconNoise sets a threshold number of flows, while length screening in the standard pipeline sets a threshold number of bases. Error correction or altered base calling can also change the fate of sequences during subsequent quality screening, such as by eliminating ambiguous bases that would otherwise result in culling of a read.

**Figure 3:**
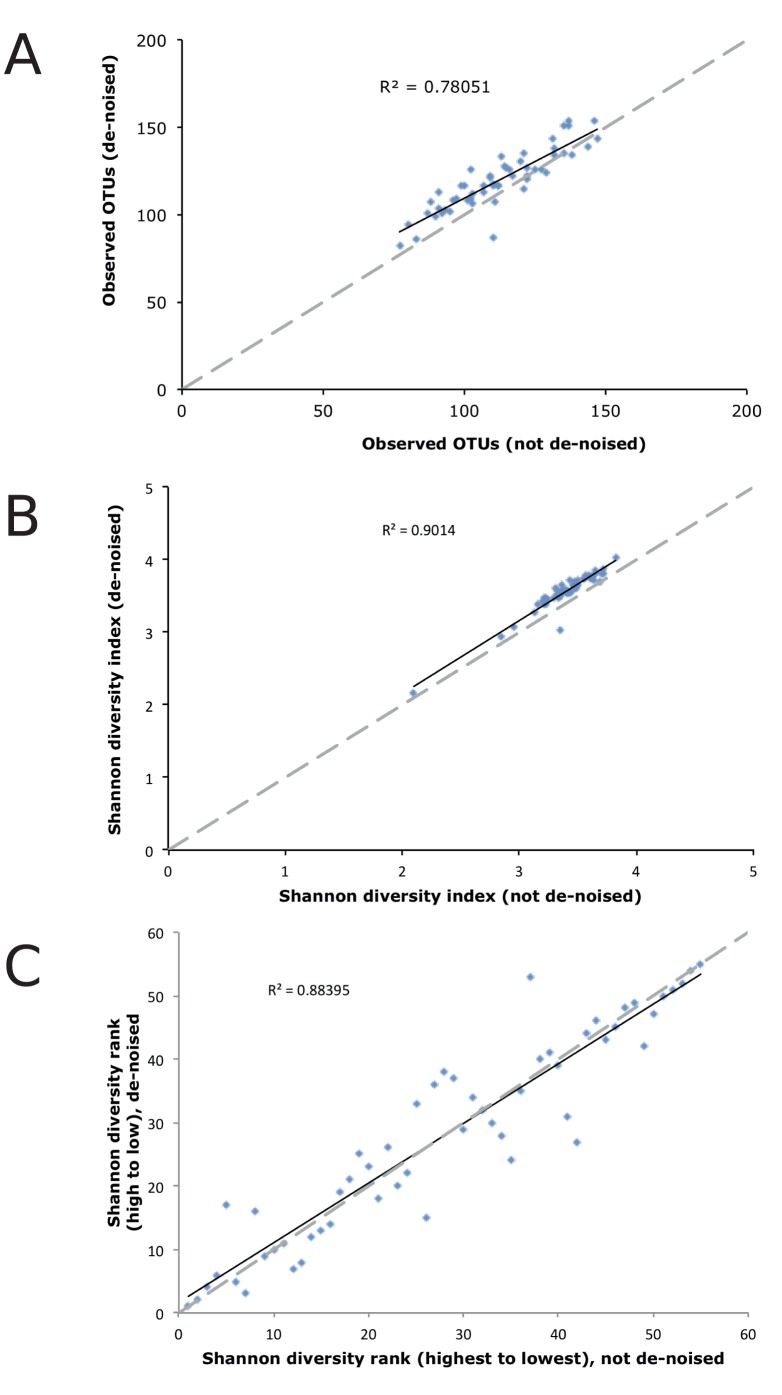
Impacts of de-noising on OTU richness and diversity. A) Relationship between OTU richness with and without de-noising, by sample. **B)** Relationship between OTU diversity (Shannon index) with and without de-noising, by sample. **C)** Ranking of samples by Shannon diversity index with and without de-noising.

Among the 232,792 sequence reads that were output after de-noising, 78.5% were unchanged by the AmpliconNoise algorithm. Among sequences that were changed by the algorithm, the average implied error rate was 1.51%, equal to approximately 1.2 corrections per sequence. “Implied error rate” indicates that, for this dataset derived from unknown organisms, we cannot know whether the changes made during de-noising correspond to true sequencing or PCR errors. The uneven division of implied errors across sequence reads is in keeping with previous reports that pyrosequencing errors tend to be clustered in a subset of sequence reads [2]. The overall error rate implied by AmpliconNoise processing for our dataset was 0.17%, which is comparable to published estimates of pyrosequencing error rates (0.12% to 0.50%; [2], [14], [19]). Variation in implied error rates across our samples was modest, ranging from 0.11% to 0.24%.

A partial explanation for the variability in error rates may relate to differences in community composition, since the frequency and length of homopolymeric runs are known to impact error rates in 454 pyrosequencing [14]. Samples containing more homopolymeric runs did have higher implied error rates (Figure S1; r^2^ = 0.17, p = 0.002). Samples containing a higher number of reads which were culled on quality score in the standard pipeline also had higher implied error rates in the AmpliconNoise pipeline (Figure S2; r^2^ = 0.10, p = 0.018). However, the range of implied error rates across our samples may also reflect a sensitivity of AmpliconNoise to differences in the structure of the input sequence set. For instance, more diverse communities had higher AmpliconNoise implied error rates than low diversity communities (Figure S3; r^2^ = 0.22, p<0.001). Additional factors may also have contributed to variation in error rates. For instance, humic substances from soil can inhibit nucleic acid amplification [20], and unequal concentrations or degree of removal of humic substances among samples may have impacted amplification efficiency or fidelity. Finally, it is possible that some of the implied error in this dataset was due to legitimate sequence variants that were removed by AmpliconNoise processing.

**Table 2:** ANOVA results tables for tests for differences in OTU diversity among two main treatment effects.

OTU diversity (Shannon index) ∼ Plant richness * Host species						
**AmpliconNoise processed**						
Source	DF	Sum of Squares	Mean Square	F Value	Pr>F	
Model	19	2.66	0.14	3.35	0.0009	**
Error	35	1.46	0.042			
Corrected Total	54	4.12				
	DF	Type III SS	MS	F	Pr>F	
Richness	4	1.17	0.29	6.99	0.0028	**
Species	3	0.38	0.13	3.02	0.042	*
Diversity*Species	12	1.53	0.13	3.07	0.0048	**
**Standard processed**						
**Source**	**DF**	**SS**	**MS**	**F**	**Pr>F**	
Model	19	1.78	0.094	1.74	0.077	.
Error	35	1.89	0.054			
Corrected Total	54	3.67				
	**DF**	**Type III SS**	**MS**	**F**	**Pr>F**	
Richness	4	0.76	0.19	3.53	0.016	*
Species	3	0.32	0.11	2.01	0.13	
Diversity*Species	12	0.89	0.074	1.37	0.22	

We contrast the significance of differences when the underlying data is processed through AmpliconNoise (top) vs. through a standard pipeline (bottom).

### OTU-based Microbial Community Analysis

When characterizing complex and unknown communities by DNA sequencing, it is common to bin sequences into OTUs based on a simple sequence similarity threshold. Processing methods will impact the conclusions reached in such studies only if they change patterns of sequence grouping during OTU formation. As expected, de-noising reduced the total number of OTUs observed across the dataset as a whole (Table 1). This was due more to differences in read retention than to impacts of error correction on clustering of reads. Of the 1,166 OTUs formed by the standard pipeline, 397 consisted entirely of reads that had been culled by AmpliconNoise (Figure 1A). In turn, AmpliconNoise processing generated 53 OTUs consisting entirely of reads that had been culled in the standard pipeline (Figure 1A). This loss of rare OTUs is the expected outcome of de-noising. Interestingly though, much of the reduction in OTU richness achieved by AmpliconNoise can be attributed to read culling rather than to error correction; considering only shared reads, AmpliconNoise processing reduced OTU richness by just 4% (Table 1). In most cases, clearly corresponding OTUs could be identified between datasets (Figure 1B). For 79% of AmpliconNoise OTUs, all shared reads were located in a single corresponding OTU in the standard pipeline dataset, and for only 5% of AmpliconNoise OTUs were shared reads distributed across more than three standard OTUs.

The rank-abundance distribution of OTUs generated by the two methods differed significantly (Figure 2; two-sample Kolmogorov-Smirnov test; D = 0.20, p<0.0001). Interestingly, de-noising did not increase the relative abundance of the most common OTUs. Rather, the membership of the most abundant OTUs was reduced while the membership of OTUs of intermediate abundance was increased (Figure 2) relative to the standard pipeline. Evenness was higher for the de-noised data than for the standard data (Shannon evenness of 0.64 vs. 0.58 overall; 1.72 vs. 1.66 per sample, t_paired_ = −13.81, p<0.001). Altered rank-abundance distributions as a result of denoising have not received explicit attention, but have great potential to impact the conclusions that are drawn from datasets.

**Figure 4:**
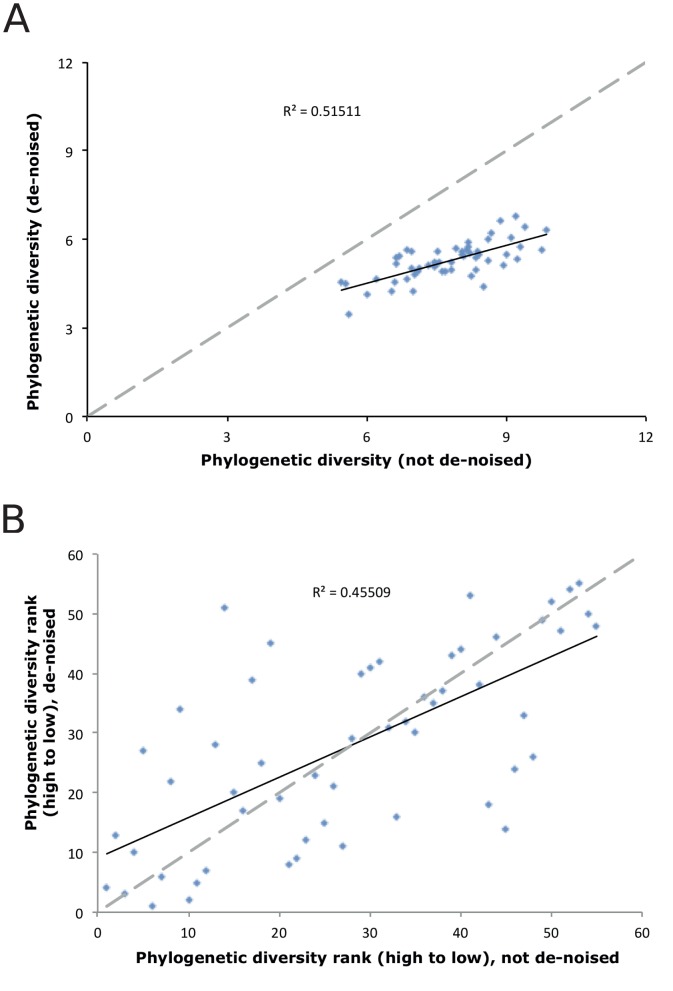
Impacts of de-noising on phylogenetic diversity. A) Relationship between phylogenetic diversity with and without de-noising, by sample. **B)** Ranking of samples by phylogenetic diversity with and without de-noising.

For instance, it is now recommended that data be rarified in order to equalize sampling effort across samples. The OTU abundance distribution will influence the effects of this procedure on subsequent analyses; the greater the proportion of rare OTUs in a dataset, the more likely that OTUs will be lost while rarifying. After rarifying our dataset, OTU richness was consistently and significantly greater on a per sample basis in the de-noised dataset (Figure 3A; t_paired_ = −6.69, p<0.001). This is exactly the opposite of the impacts of denoising on the overall richness of the dataset as a whole. Estimated OTU richness (Chao estimator) was impacted more variably by de-noising (Figure S4): communities with smaller OTU richness generally had increases in estimated OTU numbers following de-noising, while communities having greater OTU richness had declines in OTU numbers following de-noising. Richness estimates are heavily influenced by low-abundance OTUs, and their use for molecular diversity surveys has been questioned [21].

Shannon diversity estimates were also significantly higher after de-noising than with standard processing (Figure 3B; t_paired_ = −14.08, p<0.001), probably due to a combination of increased per sample OTU richness and increased evenness. Processing through AmpliconNoise sometimes markedly shifted the ranking of samples according to diversity index value (Figure 3C), which is more significant for biological interpretation than consistent increases or decreases in index values. When testing for differences in OTU diversity among experimental treatments, we observed both a greater number of significant treatment effects and more highly-significant effects following AmpliconNoise processing compared to the standard pipeline (Table 2). These pipeline-based differences in analytical outcome suggest distinct biological conclusions about the factors that influence microbial community composition. Notably, an enhanced ability to distinguish among experimental treatments provides implicit support for the AmpliconNoise processing pipeline as being useful in reducing experimental ‘noise.’

### Phylotype-based Microbial Community Analysis

There are shortcomings associated with binning DNA sequences into OTUs, and complementary approaches have been developed that use phylotype methods for analyzing microbial community DNA sequences. These approaches often map sequence reads onto large dendrograms and analyze resulting branch lengths between sequences from each sample [22]. It is expected that these methods may be less sensitive to sequence errors because typically only short branches will be introduced to the phylogenetic tree [23]. However, we saw more dramatic effects of de-noising on phylogenetic diversity than on OTU diversity. Phylogenetic diversity was reduced after de-noising (Figure 4A; t_paired_ = −24.65, p<0.001), which is the expected outcome. However, the ranking of samples according to phylogenetic diversity switched without any apparent pattern (Figure 4B). This was unexpected, and could dramatically impact comparisons among samples. Further studies contrasting the impacts of de-noising on phylogenetic estimates of microbial diversity among communities differing in structure and composition are needed to determine the generalizability of these findings.

### Conclusions

Although the rapid turnover of techniques used in microbial community analysis provides a disincentive for researchers to invest time and energy in careful evaluation of specific data processing methods, it is vital that such studies are undertaken. All methodologies are likely to contain unexpected effects that may only become clear after detailed comparisons across methods in many different datasets. Such unexpected effects should be revealed quickly, as methodological impacts on the conclusions of studies can be carried into the literature and few datasets are ever rigorously re-evaluated with updated methodologies. Furthermore, detailed analysis of data processing methods can inform refinements to algorithms and may demonstrate the need for entirely new data handling procedures.

To date, the criteria for evaluating processing methods for pyrosequence data have largely focused on the ability to successfully recreate the correct number of OTUs. This criterion does not account for other effects that may accompany the choice of data processing methods. Thankfully, more nuanced impacts, such as changes in the relative abundance of certain taxa [24], are beginning to be considered in evaluating methods. We have provided a template for evaluation that provides more detail on the effects of data processing methods on biological conclusions, extending to the full story of testing hypotheses and drawing interpretations in an actual, complex experimental design. This is a dramatically different approach that offers complementary insights to those gained from use of defined mock communities.

Our data support a positive effect of denoising in reducing spurious diversity. For instance, the AmpliconNoise pipeline eliminated hundreds of OTUs which were formed by the standard pipeline, and enhanced our ability to differentiate among experimental treatments. However, we highlight the unexpected outcome of denoising leading to higher per sample richness and diversity estimates. This is an important caution against assuming that adjustments to data processing methods will have simple outcomes (ie. that de-noising sequence data will lead to reductions in richness estimates).

The microbial ecology research community faces significant challenges in contrasting and synthesizing analyses performed over time. There is a need for ongoing re-processing and analysis of existing datasets in order to establish legitimate comparisons with more recently collected data, since the choice of data processing pipelines carries significant interpretive impacts. This work also emphasizes the importance of archiving pre-processed data as a reference for future analyses.

## Supporting Information

Figure S1 **Relationship between the presence of homopolymeric runs and AmpliconNoise implied error rate, by sample.**
(TIFF)

Figure S2 **Relationship between proportion of reads culled on quality score and AmpliconNoise implied error rate, by sample.**
(TIFF)

Figure S3 **Relationship between OTU diversity and AmpliconNoise implied error rate, by sample.**
(TIFF)

Figure S4 **Relationship between OTU richness (Chao estimate) with and without de-noising, by sample.**
(TIFF)
