# Supplementary figures and images for: Implications of Pyrosequencing Error Correction for Biological Data Interpretation
Source: PLoS One. 2012 Aug 30;7(8):e44357. doi: 10.1371/journal.pone.0044357 (PMC3431371; doi:10.1371/journal.pone.0044357)

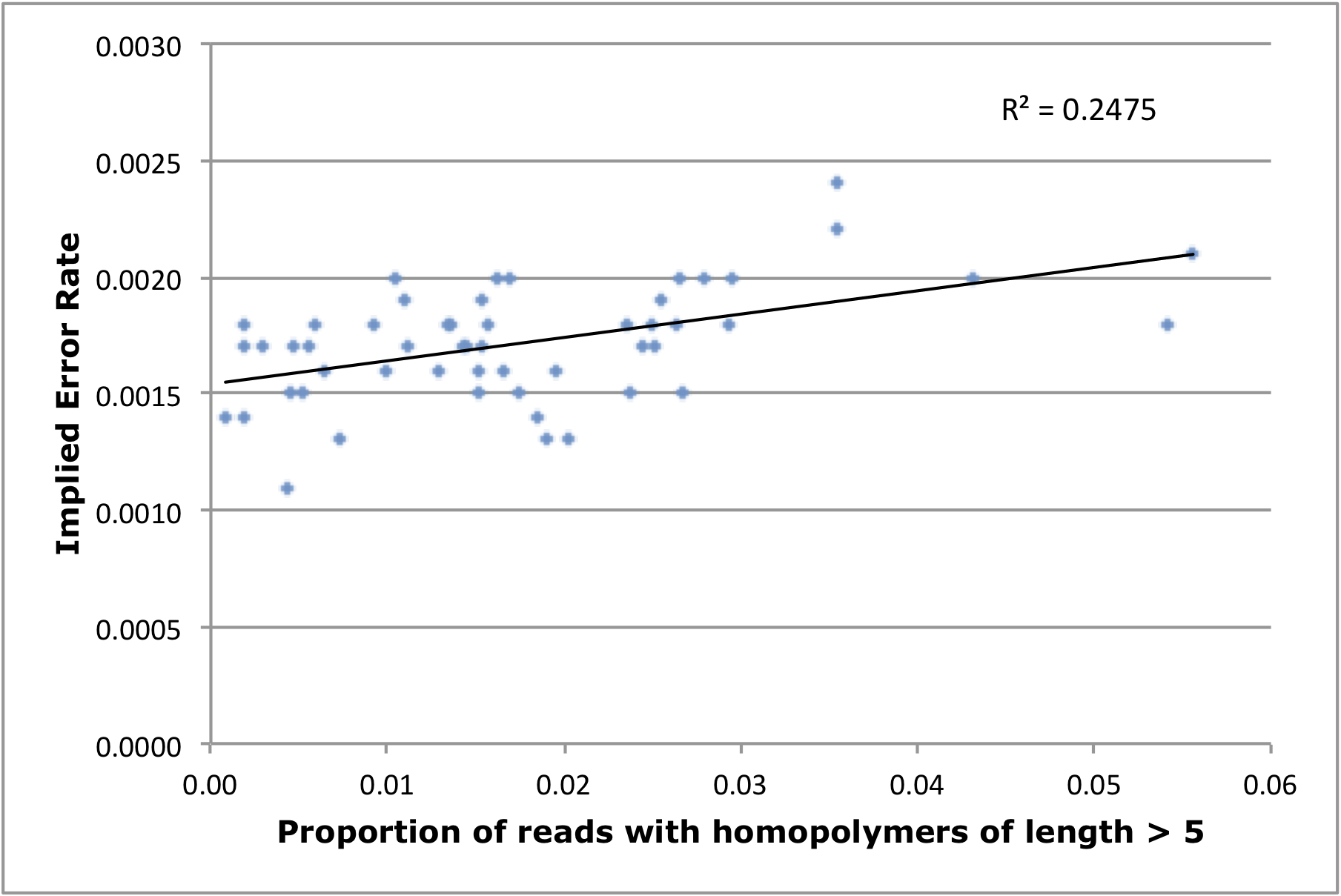

Supplement: Figure S1 — Relationship between the presence of homopolymeric runs and AmpliconNoise implied error rate, by sample. (TIFF) [file pone.0044357.s001.tiff]

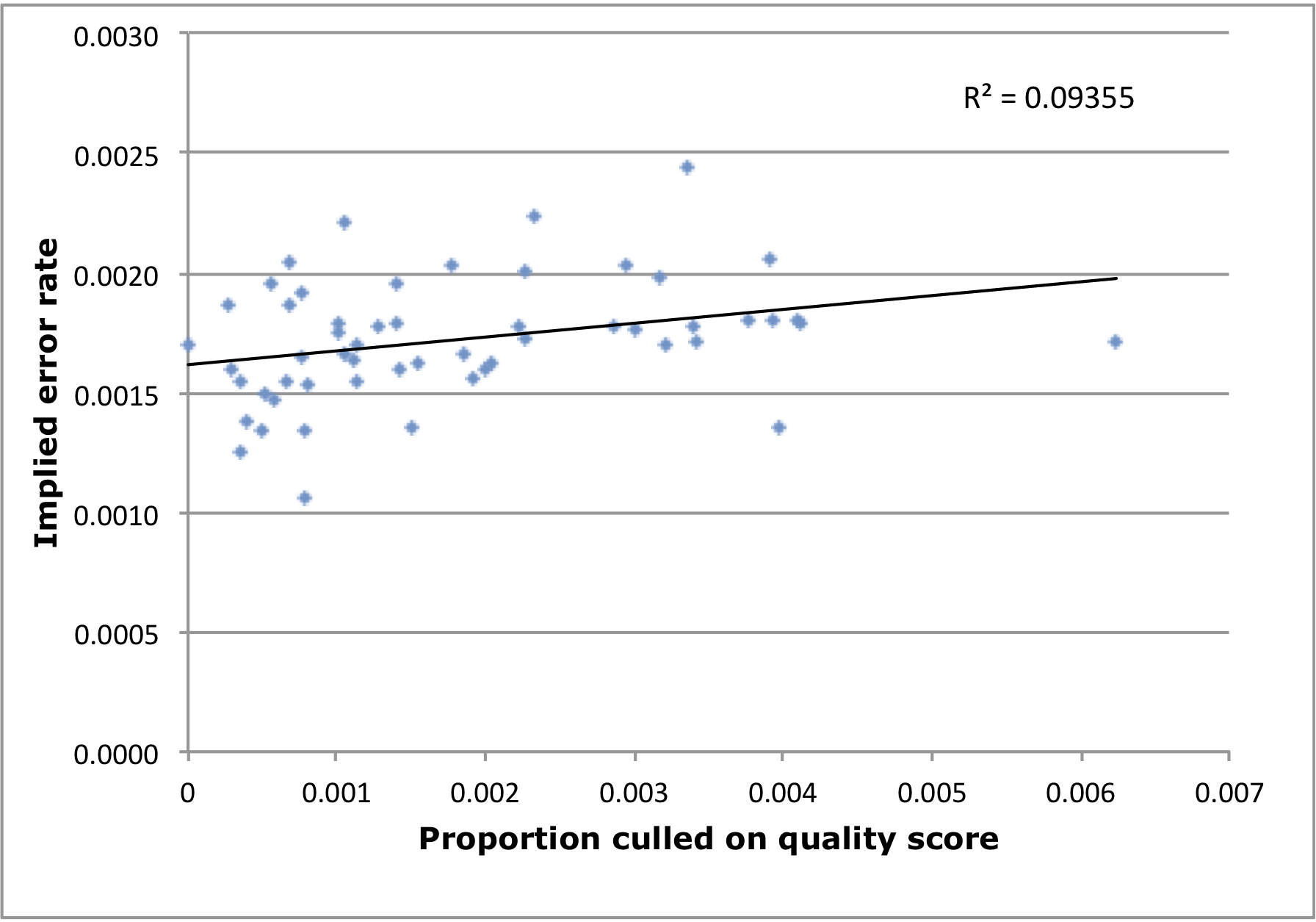

Supplement: Figure S2 — Relationship between proportion of reads culled on quality score and AmpliconNoise implied error rate, by sample. (TIFF) [file pone.0044357.s002.tiff]

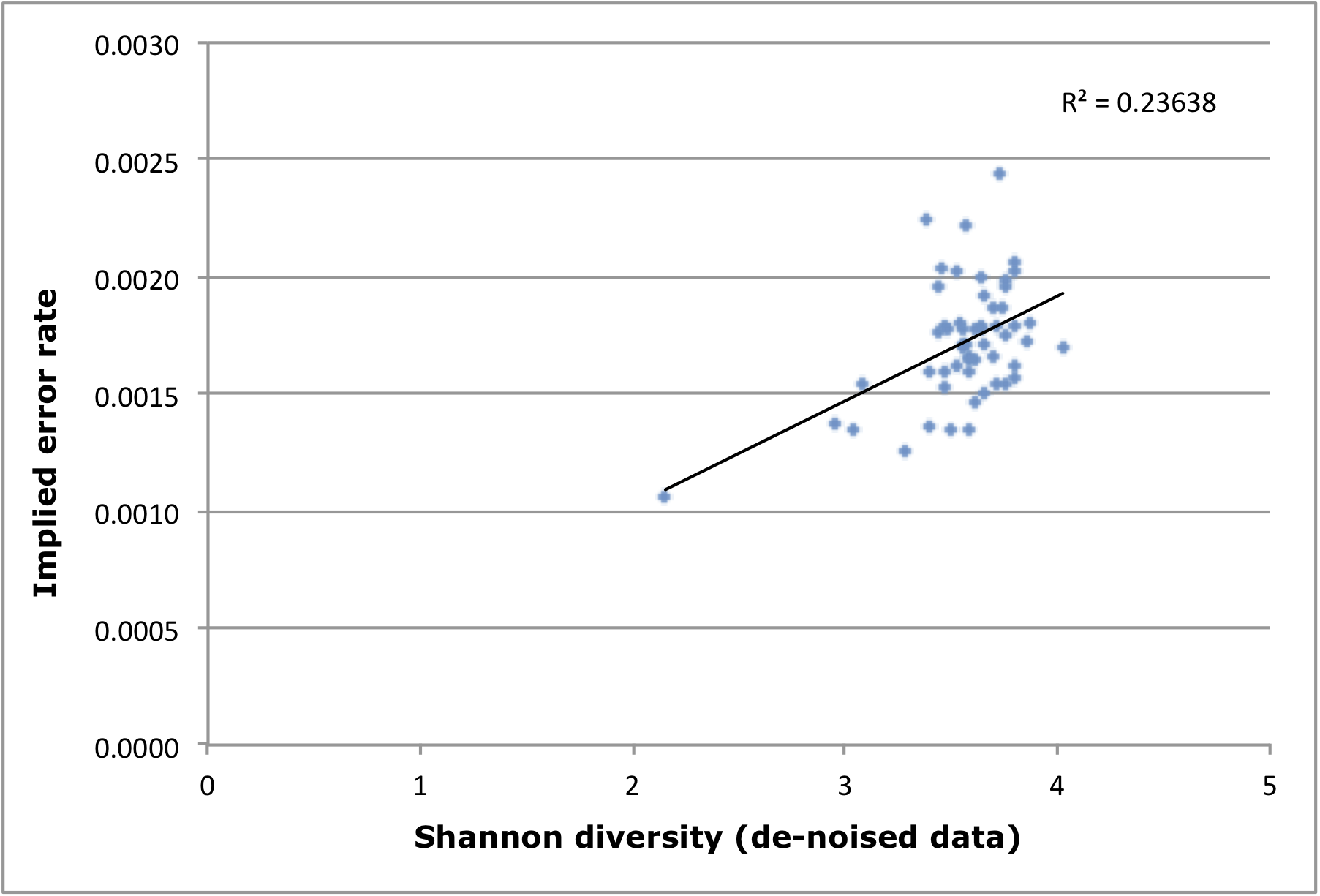

Supplement: Figure S3 — Relationship between OTU diversity and AmpliconNoise implied error rate, by sample. (TIFF) [file pone.0044357.s003.tiff]

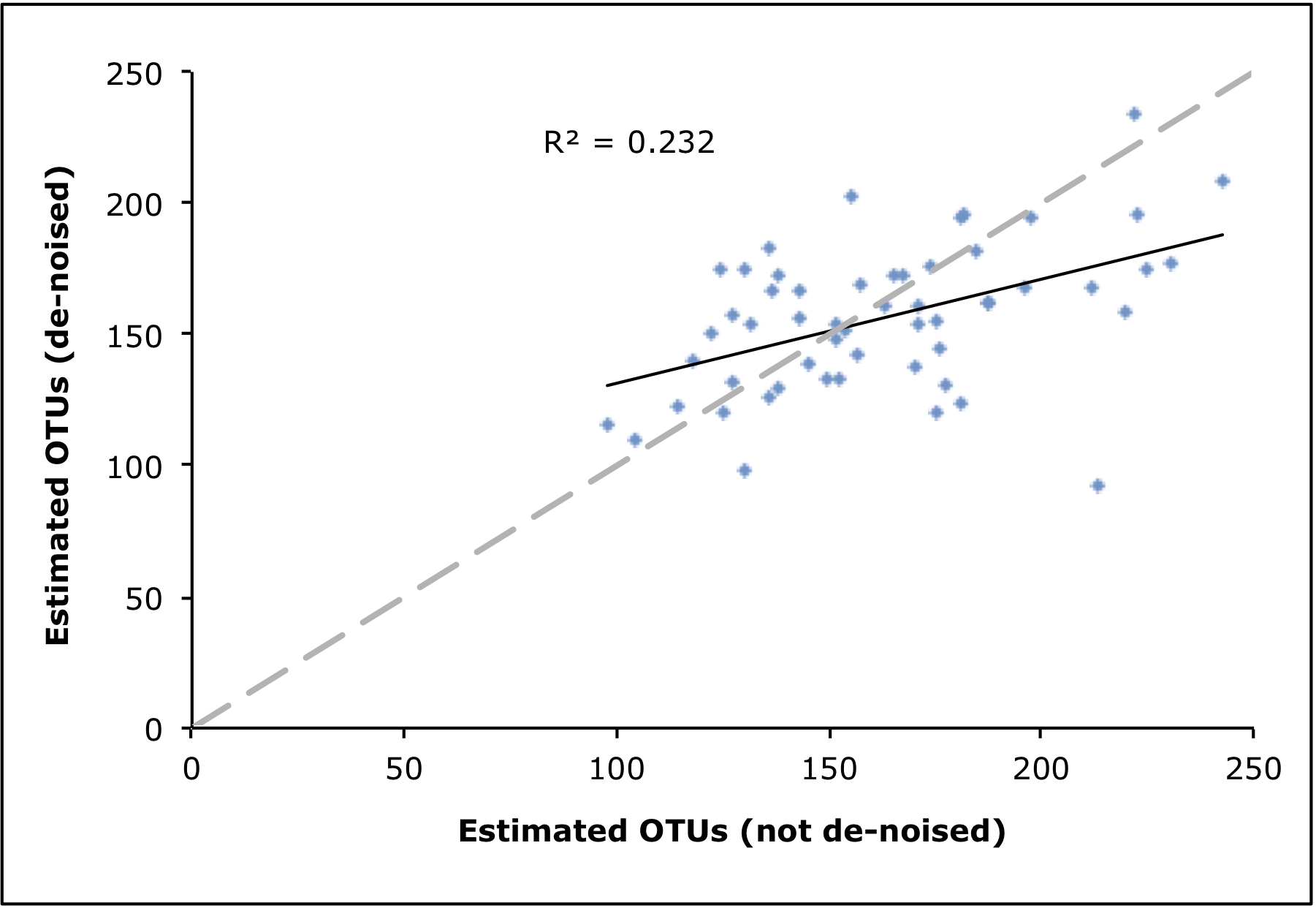

Supplement: Figure S4 — Relationship between OTU richness (Chao estimate) with and without de-noising, by sample. (TIFF) [file pone.0044357.s004.tiff]
